# Inhibition of Contractile Function in Human Joint Capsule Myofibroblasts by Targeting the TGF-β1 and PDGF Pathways

**DOI:** 10.1371/journal.pone.0145948

**Published:** 2016-01-05

**Authors:** Stefan G. Mattyasovszky, Jochen Wollstädter, Anne Martin, Ulrike Ritz, Andreas Baranowski, Christian Ossendorf, Pol M. Rommens, Alexander Hofmann

**Affiliations:** Department of Orthopaedics and Traumatology, University Medical Centre of the Johannes Gutenberg-University of Mainz, Mainz, Germany; University of Sheffield, UNITED KINGDOM

## Abstract

**Background:**

Contractile myofibroblasts (MFs) accumulate in the joint capsules of patients suffering from posttraumatic joint stiffness. MF activation is controlled by a complex local network of growth factors and cytokines, ending in the increased production of extracellular matrix components followed by soft tissue contracture. Despite the tremendous growth of knowledge in this field, inconsistencies remain in practice and prevention.

**Methods and Findings:**

In this *in vitro* study, we isolated and cultured alpha-smooth muscle actin (α-SMA) positive human joint capsule MFs from biopsy specimens and investigated the effect of profibrotic and antifibrotic agents on MF function. Both TGF-β1 and PDGF significantly induced proliferation and increased extracellular matrix contraction in an established 3D collagen gel contraction model. Furthermore, both growth factors induced α-SMA and collagen type I gene expression in MFs. TGF-β1 down-regulated TGF-β1 and TGF-β receptor (R) 1 and receptor (R) 2 gene expression, while PDGF selectively down-regulated TGF-β receptor 2 gene expression. These effects were blocked by suramin. Interestingly, the anti-oxidant agent superoxide dismutase (SOD) blocked TGF-β1 induced proliferation and collagen gel contraction without modulating the gene expression of α-SMA, collagen type I, TGF-β1, TGF-β R1 and TGF-β R2.

**Conclusions:**

Our results provide evidence that targeting the TGF-β1 and PDGF pathways in human joint capsule MFs affects their contractile function. TGF-β1 may modulate MF function in the joint capsule not only via the receptor signalling pathway but also by regulating the production of profibrotic reactive oxygen species (ROS). In particular, anti-oxidant agents could offer promising options in developing strategies for the prevention and treatment of posttraumatic joint stiffness in humans.

## Introduction

Post-traumatic joint stiffness primarily occurs after fractures and dislocations of the upper extremity with articular involvement and is a common problem for orthopaedic and trauma surgeons [1–4]. Joint stiffness is associated with soft tissue swelling, shortening of extracellular matrix fibres, and scar tissue formation. The adhesion of capsulo-ligamentous structures to the underlying bone results in loss of motion in the affected joint [5]. The healing of injured soft tissues is a dynamic process characterized by cell recruitment, migration, proliferation, differentiation, synthesis of extracellular matrix (ECM), and tissue remodelling [6–9]. Post-traumatic joint stiffness is characterized by elevated numbers of myoblastically-differentiated fibroblasts, the so-called myofibroblasts (MFs), in the capsule [10, 11]. MFs may originate from both local connective tissues and other precursor cells [12]. A hallmark of the myofibroblast phenotype is the expression of alpha-smooth muscle actin (α-SMA) and the potential to contract the surrounding ECM [13–16].

The transition from fibroblast to MF is regulated by mechanical stress, transforming growth factor-beta 1 (TGF-β1) and fibronectin (ED-A splice variant) [17, 18]. In this context, it is important to note that MFs may not be terminally differentiated after their recruitment and activation. Studies revealed that MFs reverse their phenotypes into less-active fibroblasts after treatment with appropriate cytokines, e.g., fibroblastic growth factor (FGF) or heparin [19]. At the end of physiological wound healing, MFs usually disappear via apoptosis [12, 20]. In our previous study, we focused on the effect of the pro-inflammatory cytokine tumour necrosis factor-alpha (TNF-α) on the cellular functions of human joint capsule MFs [16]. TNF-α significantly inhibits extracellular matrix contraction in a dose-dependent manner by down-regulating α-SMA and collagen type I gene expression in MFs. This effect is specifically prevented by the application of the TNF-α inhibitor infliximab and partially reduced by the COX2 inhibitor diclofenac. Despite tremendous growth of knowledge in this field over the past decade, the underlying mechanisms of posttraumatic joint stiffness that may offer new targets that interfere with excessive scar tissue formation are still poorly understood [5]. A recent study reported the absence of MFs in human elbow capsule more than five months after trauma, and there is still controversy over whether post-traumatic joint stiffness is strictly linked to the long-standing presence of MFs [21]. However, MFs likely remain in an active status under certain circumstances. A complex interaction of different growth factors, cytokines, and adhesion molecules may create an environment that triggers the prolonged MF proliferation and excessive scar formation with high ECM turnover representative of fibroconnective disorders [22]. TGF-β1 and the platelet-derived growth factor (PDGF) families of growth factors are key factors in the fibrotic response. They play pivotal roles in stimulating the replication, survival, and migration of MFs in the pathogenesis of fibrotic disorders [23, 24]. These findings need further evaluation in the context of post-traumatic joint stiffness, as the effect of these cytokines may be both site- and organ-specific.

The aim of the present study was to evaluate the effect of potential MF inhibitors (suramin, superoxide dismutase (SOD), and TGF-β1 antibody) on the functional activities of human joint capsule MFs *in vitro*. Based on the current data, we hypothesized that these substances modulate TGF-β1- and PDGF-mediated cell responses in human joint capsule MFs and may prevent ECM contraction.

## Materials and Methods

### Cell isolation and *in vitro* cultivation of human joint capsule MFs

Human joint capsules were obtained from 14 adult patients (8 women, 6 men) with a mean age of 60 years (range 23 to 84) undergoing orthopaedic or reconstructive trauma surgery. In detail, the diagnoses were advanced osteoarthritis of the hip (n = 6) treated with hemi or total hip arthroplasty, advanced osteoarthritis of the knee (n = 3) treated with total knee arthroplasty, and proximal humeral fractures (n = 2) and elbow fractures (n = 3) treated with open reduction and internal fixation (ORIF). The patients included in our study were neither operated on before nor suffered from rheumatic diseases. The joint capsules used for the study were considered to be surgical waste and would otherwise have been discarded by the hospital. All experiments were approved by the local ethic commission of Rheinland Pfalz [RLP 837.109.05(4767)], and written informed consent was obtained from every participating patient.

The joint capsule biopsies were minced with a sterile scalpel and processed within 6 hours after surgical excision. The inner layer of the capsule, the synovial membrane, which was loosely attached to the external fibrous capsule was carefully dissected from the fibrous tissue. For all experiments, the outer layer of the joint capsule with the fibrous tissue was used. The samples were rinsed in phosphate-buffered saline (PBS, Dulbecco’s Phosphate Buffered Saline, Invitrogen) to remove blood and fat residues and gradually digested in a water bath at 37°C with a mixture of type IV collagenase (1 mg/ml, Sigma-Aldrich Chemie GmbH, Steinheim, Germany), trypsin (2.5 mg/ml, Sigma-Aldrich Chemie GmbH, Steinheim, Germany), and DNase I (2 mg/ml, Applichem GmbH, Darmstadt, Germany). The specimens were filtered through a cell strainer (100 μm mesh, BD Biosciences, Heidelberg, Germany) after 45 and 90 minutes of incubation to obtain a single-cell suspension. The cell supernatant was washed in serum-free DMEM (Dulbecco’s Modified Eagle’s Medium, Biochrom AG, Berlin, Germany) supplemented with 10,000 U/ml penicillin G sodium and 10,000 μg/ml streptomycin sulphate (Gibco Invitrogen, Karlsruhe, Germany), and finally centrifuged at 1.4x10^3^ rpm for 5 minutes at 4°C. The cell pellet was resuspended in DMEM supplemented with 10% heat-inactivated fetal calf serum (FCS, PAA laboratories, Pasching, Austria) and antibiotics, seeded into culture flasks (Cellstar, Greiner Bio-One GmbH, Frickenhausen, Germany) and incubated in a humidified atmosphere of 5% CO_2_ at 37°C. Cells were quantified using a Neubauer chamber. Culture media were changed twice a week, and preconfluent cells were passaged using accutase (PAA laboratories). Early passage cells (passages 2–4) were used for all experiments. Immunohistochemical staining and the establishment of the proliferation assay were performed with cell cultures in pre-tests before starting the experiments with growth factors and inhibitors. All individual cell cultures provided 2–8 million cells after 2–3 passages that were sufficient to perform all tests. The cells from different donors were not pooled.

### Immunohistochemical staining of the cell cultures

For MF detection, the cells were seeded on histological cover slides at a density of 25,000 cells/cm^2^, incubated for 24 h in complete cell culture medium, and fixed with 3.7% paraformaldehyde. The expression of α-smooth muscle actin (α-SMA) in MF cultures was verified using a monoclonal mouse-anti-human α-SMA antibody (DaKo, Hamburg, Germany) and a biotin-labelled rabbit-anti-mouse IgG secondary antibody (DaKo, Hamburg, Germany). Immune complexes were stained with streptavidin-HRP and 3,3´-diaminobenzidine (DAB). Cell nuclei were counterstained with Mayer’s hemalum (Merck AG, Darmstadt, Germany).

### Viability and proliferation assays in human MF cultures

MFs from human joint capsules were seeded into 96-well plates (Greiner) at a density of 25,000 cells/cm^2^ and incubated in 150 μl serum-supplemented DMEM (5% FCS, 1% penicillin/streptomycin) for 48 h. Thereafter, the cell layers were washed with PBS and incubated for 24 h in 150 μl serum-reduced DMEM supplemented with 1% FBS (27). After 24 h, the cells were washed with PBS and incubated for 72 h in 150 μl serum-free DMEM (1% bovine serum albumin, 37°C, 5% CO_2_) containing growth factors in the different concentrations listed in Table 1. Furthermore, MF proliferation was investigated under the influence of suramin, a non-specific blocker of TGF-β1 and PDGF that competitively binds to the growth factor receptors, superoxide dismutase (SOD), an inhibitor of reactive oxygen species (ROS)-mediated TGF-β gene expression, or specific TGF-β1 antibodies. Twelve different groups (A-L) were chosen in this study (Table 1). The concentrations of the investigated growth factors were used according to data from previous *in vitro* experiments [25–28]. For the negative control group A, cell cultures from 20 different individuals were established, and all measurements were performed in quadruplicate (total number of measurements n = 80). These experiments involving growth factors and/or inhibitors were performed with cells from four different individuals with four replicate measurements each (total number of measurements n = 16). Cell viability and proliferation were assessed after 96 h using the standardized colorimetric 3-(4,5-dimethylthiazol-2-yl)-2,5-diphenyl tetrazolium bromide (MTT) assay (Promega GmbH, Mannheim, Germany) determining the activity of mitochondrial succinyl dehydrogenase. After 96 h, 30 μl of 0.5% MTT solution was added to each well and incubated for 2 h. The medium was removed, and the dye was resolved with 100 μl isopropanol (Hedinger GmbH & Co. KG, Stuttgart, Germany). The optical density was measured at 570 nm (650 nm background) using an ELISA Reader (TECAN Sunrise, Crailsheim, Germany).

**Table 1:** The experimental design to study the effect of growth factors and their potential inhibitors on human joint capsule MFs *in vitro*.

Group	Growth factors / Inhibitors
A	Control group
B	TGF-β1 (0.1 ng/ml)
C	TGF-β1 (10 ng/ml)
D	PDGF (1 ng/ml)
E	PDGF (10 ng/ml)
F	SOD (500 IU/ml)
G	TGF-β1 (10 ng/ml) + SOD (500 IU/ml)
H	suramin (50 μg/ml)
I	TGF-β1 (10 ng/ml) + suramin (50 μg/ml)
J	PDGF (10 ng/ml) + suramin (50 μμg/ml)
K	TGF-β1 antibody (5 μg/ml)
L	TGF-β1 (10 ng/ml) + TGF-β1 antibody (5 μg/ml)

GF-β1: transforming growth factor-β1; PDGF: platelet derived growth factor; SOD: superoxide dismutase.

### Three-dimensional (3D) collagen type-1 gel contraction assay

A three-dimensional (3D) collagen type-1 gel contraction assay was used to estimate the contractile forces exhibited by MFs as described previously [16]. Collagen type-1 gel is permeable to relevant nutrients and growth factors. Collagen gels were prepared using type I rat collagen (1.5 mg/ml, BD Biosciences, Bedford, USA) in a 10-fold Medium 199 concentrate, 7.5% NaHCO_3_, 1N NaOH (all from Sigma-Aldrich, Steinheim, Germany), and distilled water. As this model simulates the surrounding extracellular matrix (ECM) of joint capsules, MFs grow in a physiological three-dimensional environment. Moreover, differentiated MFs are able to contract the ECM, which leads to a detectable contraction. The cells were resuspended in the gel solution at a concentration of 1.5 mg/ml collagen type I and seeded into 24-well plates (four wells per group) at a density of 2x10^5^ cells/500 μl. After 30 minutes, 1 ml serum-supplemented DMEM (5% FCS) was added, and the solidified gels were incubated for 48 hours. Thereafter, the gels were carefully washed with PBS and incubated for 24 h in 1 ml of serum-free DMEM supplemented with 1% BSA. After rinsing with PBS, the cells were incubated for 72 hours with 1 ml serum-free DMEM containing growth factors as described in Table 1. MFs incubated in 150 μl serum-free DMEM only were used as negative controls (group A). In this group, MF cultures from nine different individuals with triplicate measurements each were established (total number of measurements n = 27). For groups B-L, cultures from four individuals with triplicate measurements each were used (total number of measurements n = 12). The gels were carefully detached from the culture plate with a pipette tip and cultured for further 48 hours in the same culture medium. Gel areas were scanned before and 48 hours post release using the Canon 660U scanner and quantified using ImageJ software (NCBI, National Center for Biotechnology Information, Bethesda, MD, USA) after seven days of incubation.

### Gene expression of α-SMA, collagen type I, TGF-β1, and TGF-β receptor 1 and 2

To quantify the gene expression of α-SMA, collagen 1 alpha 2, TGF-β1, and TGF-β receptors 1 and 2 in MFs under the influence of growth factors and their inhibitors as described in Table 1, total RNA was extracted and purified after seven days of culture from cells cultured in monolayers under the same conditions as the cells cultured in 3D collagen gels. Reverse transcription was performed using 2 μg of RNA, M-MuLV-reverse transcriptase, and hexamer primers (Peqlab Biotechnologie GmbH, Erlangen, Germany). Amplification was performed by PCR using Taq DNA polymerase (PeqLab, Erlangen, Germany) and specific primers (Table 2). Amplification conditions were varying cycle numbers (Table 2) with a 1 min denaturation step at 95°C, annealing temperatures ranging from 52 to 58°C (Table 2), and an extension phase for 1 min at 72°C. Electrophoresis was performed using 1.5% agarose gels containing ethidium bromide to visualize PCR products using an UV light imager. Densitometric quantification was performed using the 15 Vilber Lourmat Bioprofile E-Capt 12.4 system. Measurement values were indicated as fold expression of the housekeeping gene GAPDH. All PCR measurements were performed on cell cultures from four different individuals with triplicate measurements each (total number of measurements n = 12).

**Table 2:** List of primers used for reverse transcription in PCR.

primer	cycle number	annealing temperature in [°C]	product in [bp]
**GAPDH** 5´-cgt ctt cac cac cat gga ga 3´-cgg cca tca cgc cac agt tt	27	58	300
**α-SMA** 5´-gct cac gga ggc acc cct gaa 3´-ctg ata gga cat tgt tag cat	35	52	569
**TGF-β1** 5´-tgg cga tac ctc agc aac c 3´-gtt ggc atg gta gcc ctt g	35	56	426
**Collagen I alpha 2** 5´-gac atg ctc agc ttt gtg ga 3´-cct gtg gtc caa caa ctc ct	40	52	490
**TGF-β receptor 1** 5´-gag cat gga tcc ctt ttt ga 3´-aac atc gtc gag caa ttt cc	35	52	396
**TGF-β receptor 2** 5´-ata gga ctg ccc atc cac tg 3´-gct gat gcc tgt cac ttg aa	35	54	445

GAPDH: glyceraldehyde 3-phosphate dehydrogenase; TGF-β: transforming growth factor-beta; α-SMA: alpha-smooth muscle actin.

### Statistical analysis

For statistical analysis, the IBM SPSS Statistics software version 22 was used. The data distribution was defined by medians ± quartiles. The measurement values were normalized to the median of the control group (control median) and presented as % values related to the control group in box-plots. For multiple comparisons, the paired non-parametric Wilcoxon test was performed. Differences were considered to be statistically significant for p<0.05 and depicted by *p<0.05, **p<0.01, and ***p<0.001.

## Results

### MFs isolated from human joint capsules express intracellular α-SMA *in vitro*

**Fig 1:**
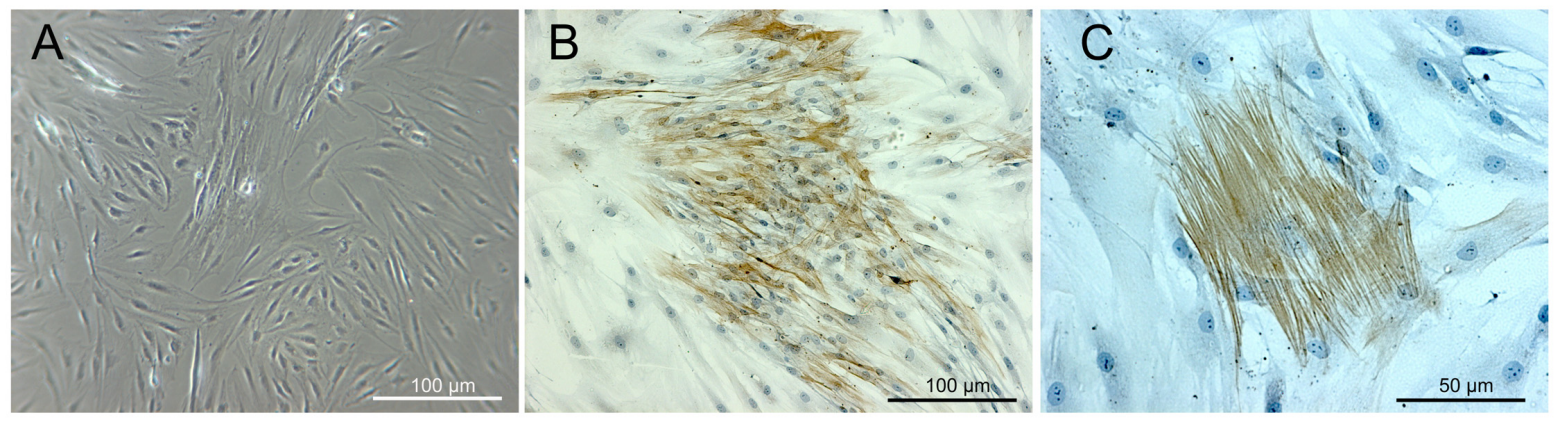
MFs isolated from human joint capsules used in this study: Morphology and intracellular expression of the marker alpha-smooth muscle actin (α-SMA). Differentiated MFs in native cell cultures were characterized by a typical flattened morphology, becoming confluent during culture (A). Immunohistological staining for the MF cell marker α-SMA in confluent cell cultures (B-C) in the form of stress fibres (C). Scale bars = 100 μm in A and B; bar = 50 μm in C.

A few days after seeding, differentiated MFs appeared as plainly spread and radiated cells with multiple cell foci. The number of these cells increased continuously during culture, rapidly becoming confluent (Fig 1A). At the beginning of the experiments, almost 100% of the confluent cell cultures stained positive for α-SMA (Fig 1B). Immunohistological staining for α-SMA, a hallmark of MF differentiation, revealed the typical appearance of cells with intracellular stress fibres (Fig 1C).

### The proliferative effect of TGF-β1 and PDGF on human joint capsule MFs is inhibited by suramin and superoxide dismutase (SOD)

**Fig 2:**
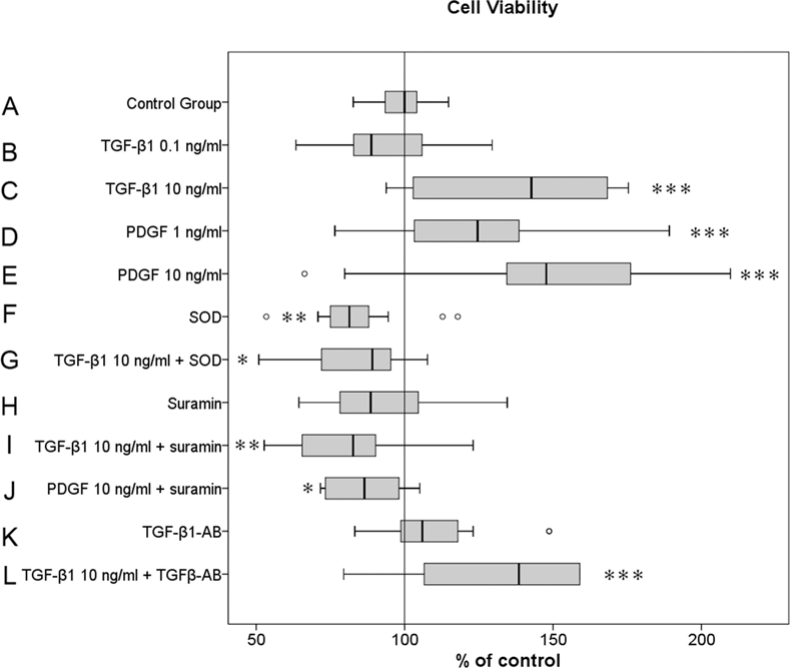
Cell viability and proliferative capacity of MFs upon stimulation with TGF-β1 and PDGF with or without the inhibitors suramin, SOD or TGF-β1 antibody. The effect of TGF-β1 (0.1 and 10 ng/ml) and PDGF (1 and 10 ng/ml) with or without the growth factor inhibitors SOD (500 IU/ml), suramin (50 μg/ml) or the TGF-β1 antibody (5 μg/ml) on myofibroblast cell viability. Data are representative of twenty experiments with cells from twenty different individuals for the control group A and from four individuals for the groups B to L with four replicate measurements from each individual patient sample (n = 80 for the control, n = 16 for all other groups). The results are plotted as % of the median in the control group according to the paired non-parametric Wilcoxon test used for the statistical analysis. *p<0.05, **p < 0.01, ***p < 0.001. MTT: 3-(4,5-dimethylthiazol-2-yl)-2,5-diphenyl tetrazolium bromide.

Proliferation assays revealed a significant and dose-dependent increase of cell viability and MF proliferation in the presence of TGF-β1 and PDGF (Fig 2).

Whereas MFs cultured with lower concentrations of TGF-β1 (group B: 0.1 ng/ml) showed no significant differences in proliferation and cell viability compared with the control group A (p = 0.12), MF proliferation was significantly (p<0.001) enhanced by 24.1% with the higher concentration of TGF-β1 (group C: 10 ng/ml). In contrast to TGF-β1, PDGF significantly enhanced MF proliferation by 29.5% (p<0.001) at the lower concentration (group D: 1 ng/ml PDGF) compared to the control group A. The most significant proliferative effect on MFs was detected in the culture with higher concentrations of PDGF (group E: 10 ng/ml), with an enhancement in cell proliferation by 57.3% above the median value level of the control group A (p<0.001). Due to the dose-dependent proliferation of MFs, the following inhibitor experiments were tested with the higher concentrations of TGF-β1 and PDGF to obtain the most obvious effects.

In MF cultures, the presence of the inhibitors SOD and TGF-β1 (group G) significantly reduced cell proliferation to 89.2% (p<0.05), meaning that the cell proliferation was significantly reduced below the median cell proliferation level of control group A. These results might be explained by the fact that SOD itself (group B: 500 IU/ml SOD) significantly reduced cell proliferation below the level of the control group A to 81.4% (p<0.01). This effect was even more significant (p<0.01) in MF cultures with 10 ng/ml TGF-β1 and 50 μg/ml suramin (group I). Suramin reduced TGF-β1-driven MF proliferation by 17.3% below the MF proliferation level of control group A. Even the significant proliferative effect of high concentrations of PDGF (group E: 10 ng/ml PDGF) was significantly (p<0.05) reduced by 50 μg/ml suramin (group J) below the level of the control group A to 86.5%. In contrast to SOD, suramin itself (group H: 50 μg/ml suramin) had no significant inhibitory effect on MF proliferation, although we observed a trend towards reduced MF proliferation below the level of the cell proliferation of control group A to 88.6% (p = 0.18). Interestingly, neither the TGF-β1 antibody itself (group K: 5 μg/ml TGF-β1) nor 5 μg/ml TGF-β1 antibody with 10 ng/ml TGF-β1 revealed a significant inhibitory effect on MF proliferation (Fig 2). Compared to control group A, the stimulatory effect of 10 ng/ml TGF-β1 (124.1%) was not influenced by the presence of 5 μg/ml TGF-β1 antibody and revealed a comparable MF proliferation level with 138.5%. This effect was not significant compared to the culture with 10 ng/ml TGF-β1 in group C (data not shown).

### TGF-β1 and PDGF enhance the MF contractile forces. SOD and TGF-β1 antibodies significantly inhibit TGF-β1-modulated ECM contraction, and suramin significantly inhibits TGF-β1- and PDGF-modulated ECM contraction

**Fig 3:**
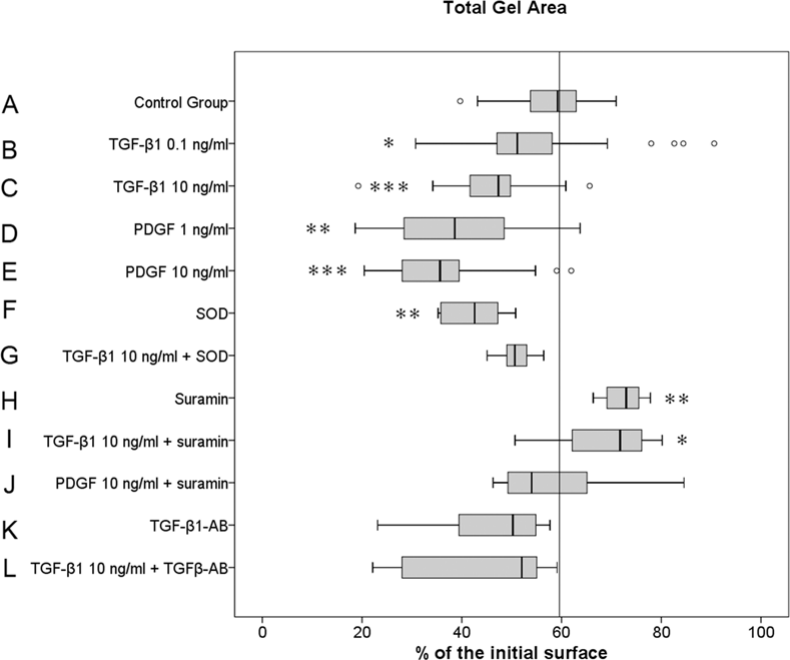
The effect of TGF-β1 and PDGF with and without SOD, suramin and TGF-β1 antibody on ECM contraction. Gel surface areas in the presence or absence of TGF-β1 (0.1 and 10 ng/ml) and PDGF (1 and 10 ng/ml) and in the presence and absence of SOD (500 IU/ml), suramin (50 μg/ml) or TGF-β1 antibodies (5 μg/ml) following the groups listed in Table 1 were scanned and calculated as described in the Materials and Methods. Data are representative of nine performed experiments with cells from nine different individuals for control group A and from four individuals for groups B to L, with triplicate measurements from each individual patient sample (n = 27 for the control, n = 12 for all other groups). The results are plotted as % of the median in the control group according to the paired non-parametric Wilcoxon test used for the statistical analysis. *p<0.05, **p<0.01, ***p<0.001.

In comparison with control group A, the addition of TGF-β1 and PDGF to 3D collagen gels significantly enhanced the dose-dependent collagen gel contraction, accompanied by a significant reduction of the gel surface area (Fig 3).

Low TGF-β1 concentration (group B: 0.1 ng/ml TGF-β1) caused a significant gel contraction to 51.1% of the initial area, being 8.5% below the median surface of the control group A (p<0.05). This effect was highly significant (p<0.001) with 10 ng/ml TGF-β1 (group C) in the 3D culture, reducing the collagen gel area 12.3% below the level of control group A. Similar to TGF-β1, PDGF also significantly enhanced gel contraction to 38.6% (p<0.001) with the lower concentration (group D: 1 ng/ml PDGF) and to 35.6% with the higher concentration (group E: 10 ng/ml PDGF), with a median reduction of the gel surface by 21% (p = 0.001) and 24% (p<0.001) below the median value level of control group A, respectively. This contractile effect of TGF-β1 was reduced by SOD (group G: 10 ng/ml + 500 IU/ml SOD) to a gel area of 50.6%, not significantly different from the median gel area of control group A. Interestingly, SOD alone (group F: 500 IU/ml SOD) significantly enhanced gel contraction with a median gel area 17% below the level of control group A (p = 0.005). In contrast to SOD, suramin alone (group H: 50 μg/ml suramin) and in 3D gel culture with 10 ng/ml TGF-β1 (group I) blocked the contractile function of MFs, increasing the gel area by 13.4% and 12.1% compared to control group A, respectively. Furthermore, suramin almost completely blocked the contractile effect of PDGF (group J: 10 ng/ml PDGF + 50 μg/ml suramin) to a median gel surface level of 54%, close to the median surface level of control group A. According to the proliferation assay results, we did not find significant effects on gel contraction by the application of the TGF-β1 antibody alone (group K), with a median gel area of 50.2%. Similarly to suramin and SOD, the TGF-β1 antibody in culture (group L) reversed TGF-β1-induced gel contraction to the level of the control group A.

### TGF-β1 and PDGF up-regulate α-SMA and collagen I gene expression in a dose-dependent manner and can be blocked by SOD and suramin

**Fig 4:**
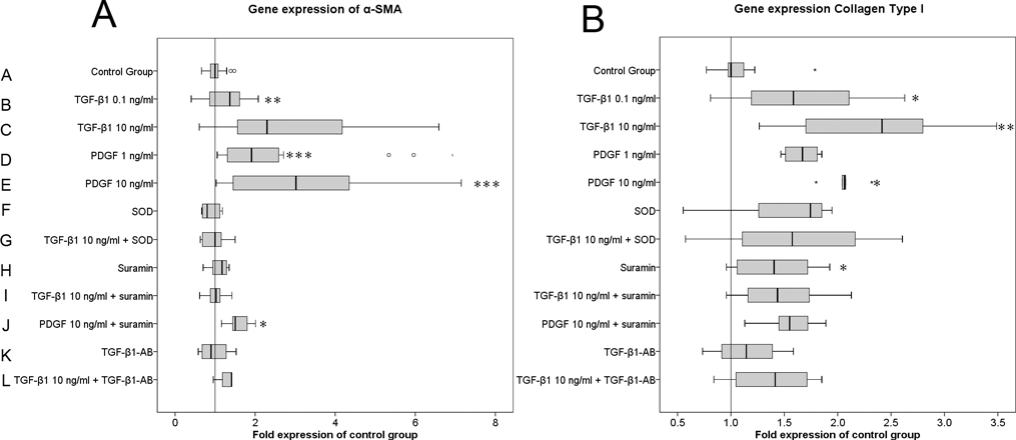
TGF-β1 and PDGF specifically up-regulate the expression of the myofibroblast marker alpha-smooth muscle actin (α-SMA) and the extracellular matrix protein collagen type I. Data are representative of four performed experiments with triplicate measurements from each individual patient sample (n = 12 for all groups). The results are plotted as fold changes of the median in the control group according to the paired non-parametric Wilcoxon test used for the statistical analysis. *p<0.05, **p<0.01, ***p<0.001.

The presence of both growth factors TGF-β1 and PDGF in the cell culture resulted in a dose dependent increase of the gene expression of the MF specific marker α-SMA (Fig 4A).

Whereas the lower concentration of TGF-β1 (group A: 0.1 ng/ml TGF-β1) had no significant effect on α-SMA gene expression (p = 0.142), the higher concentration of TGF-β1 (group C: 10 ng/ml TGF-β1) significantly induced α-SMA gene expression 2.3-fold (p = 0.001) compared to control group A. This effect was similar to stimulating the cells with PDGF, which increased α-SMA gene expression 1.9-fold (p<0.001) with 1 ng/ml and 3-fold (p<0.001) with 10 ng/ml compared to control group A. This stimulatory effect of 10 ng/ml TGF-β1 on α-SMA gene expression was almost completely blocked by SOD (group G), suramin (group I) and TGF-β1 antibody (group L) down to a level close to control group A (Fig 4A). The stimulatory effect of PDGF on α-SMA gene expression was significantly but not completely reduced 1.5-fold by suramin (group J) compared to control group A. The presence of the inhibitors in the culture alone, without any growth factors, had no effect on α-SMA gene expression.

Analogous to the α-SMA gene expression results, both TGF-β1 and PDGF up-regulated collagen I gene expression in a dose-dependent manner (Fig 4B). TGF-β1 induced a change in collagen I gene expression of 1.6-fold (p = 0.01) with 0.1 ng/ml (group B) and 2.4-fold (p<0.01) with 10 ng/ml (group C). PDGF induced collagen I gene expression 1.7-fold (p = 0.138) with 1 ng/ml (group D) and 2.0-fold (p<0.05) with 10 ng/ml (group E). The stimulatory effect of TGF-β1 was significantly down-regulated by SOD, suramin and TGF-β1 antibody (Fig 4B). Moreover, the stimulatory effect of 10 ng/ml PDGF (group J) was significantly down-regulated by suramin to the level of the control group. There was no significant effect on collagen I gene expression from the presence of SOD and TGF-β1 alone, whereas suramin (group H) significantly (1.4-fold, p<0.05) induced collagen expression compared to the control group (Fig 4B).

### TGF-β1 autoregulates TGF-β1 gene expression

**Fig 5:**
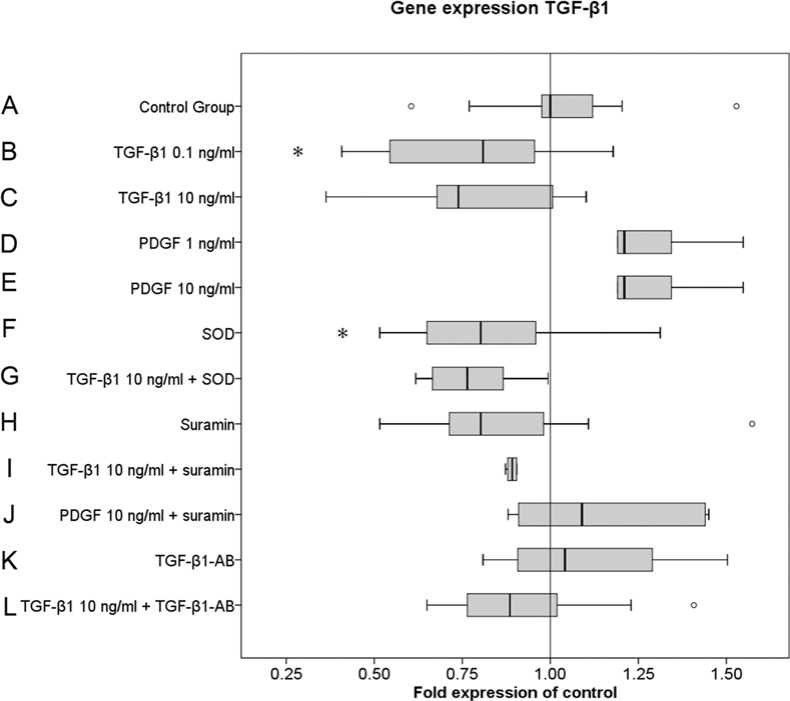
TGF-β1 down-regulates TGF-β1 gene expression. Data are representative of four performed experiments with triplicate measurements from each individual patient sample (n = 12 for all groups). The results are plotted as fold changes of the median in the control group according to the paired non-parametric Wilcoxon test used for the statistical analysis. *p < 0.05, **p < 0.01, ***p < 0.001.

MF culture with 0.1 ng/ml TGF-β1 (group B) resulted in a significant down-regulation (p<0.05) of TGF-β1 gene expression, reaching a maximum effect with 10 ng/ml TGF-β1 (group C) (Fig 5).

Although not statistically significant, we found a 1.2-fold up-regulation of TGF-β1 gene expression by PDGF (groups D and E), which was not dose-dependent. Both SOD and suramin alone down-regulated TGF-β1 gene expression. However, this effect did not reach the defined level of significance (Fig 5). None of the three inhibitors had a significant effect on TGF-β1 gene expression if cultured in the presence of 10 ng/ml TGF-β1. Furthermore, neither PDGF (group D) nor PDGF and suramin (Group J) had a significant impact on TGF-β1 gene expression.

### Gene expression of TGF-β receptors 1 and 2 is regulated by the growth factors TGF-β1 and PDGF

**Fig 6:**
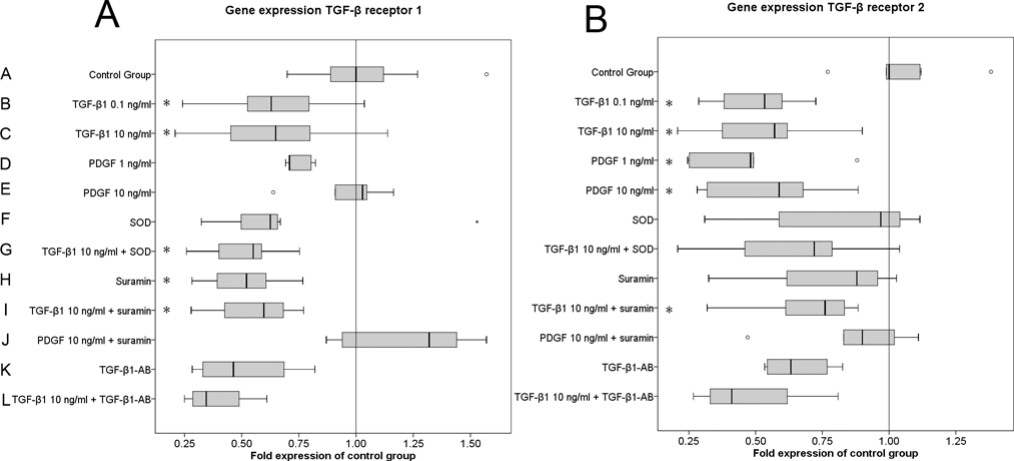
TGF-β1 down-regulates TGF-β receptor 1 and 2 gene expression, and PDGF down-regulates TGF-β receptor 2 gene expression. Data are representative of four performed experiments with triplicate measurements from each individual patient sample (n = 12 for all groups). The results are plotted as fold changes of the median in the control group according to the paired non-parametric Wilcoxon test used for the statistical analysis. *p<0.05, **p<0.01, ***p<0.001.

MF culture with TGF-β1 resulted in a significant down-regulation (p<0.05) of TGF-β receptor 1 (Fig 6A) and TGF-β receptor 2 (Fig 6B) gene expression, regardless of the concentration.

PDGF significantly down-regulated TGF-β receptor 2 gene expression (Fig 6B) (p<0.05). This effect was terminated by the addition of SOD and TGF-β1 antibody. Suramin blocked the down-regulatory effect of 10 ng/ml PDGF to the level of the control group and the down-regulatory effect of 10 ng/ml TGF-β1 by trend, but did not reach significance in our experiments. From all the tested inhibitors, only suramin alone significantly down-regulated TGF-β receptor 1 gene expression (p<0.05). Neither SOD nor the TGF-β1 antibody significantly targeted TGF-β receptor 1 (Fig 6A) or TGF-β receptor 2 (Fig 6B) gene expression, although we observed a trend towards down-regulation by all the three inhibitors (Fig 6A and 6B). Moreover, none of the tested inhibitors reversed the suppressive effect of 10 ng/ml TGF-β1 on TGF-β receptor 1 gene expression.

## Discussion

In the early stages of wound healing, undifferentiated fibroblasts migrate into the developing layer of fibrin and stabilize the surrounding tissue by the synthesis of proteoglycans and other ECM proteins. Numerous mediators and cytokines are released (e.g., TGF-β1, PDGF, FGF, EGF, VEGF, MCP, SDF-1, CTGF and interleukins) and create an environment that contributes to the activation of the messenger cells [29]. The end of the inflammatory phase is characterized by the presence of contractile MFs and the expression of collagen type I [29]. Proliferation of fibroblasts, differentiation into myofibroblasts and synthesis of ECM are elementary processes during wound healing after a substantial soft tissue injury [29, 30].

Our present knowledge of MF regulation in the development of joint capsule contracture is limited. The biological functions of MFs have been established from analyses of other pathological disorders with enhanced tissue fibrosis. In addition to physiological wound healing [30], MFs play a crucial role in fibroconnective disorders such as pulmonary and kidney fibrosis [31], cirrhosis of the liver [24], and M. Dupuytren [32]. Morbus Dupuytren presents with an unrestricted scarring and a macroscopic contracture of the palmar fascia, resulting in a progressive and irreversible flexion deformity of the fingers and finally of the whole hand. The insights from fibroconnective disorders may not reflect the fact that joint contracture particularly affects the large joints of the upper extremities. In previous studies, we found an increase in the numbers of MFs in joint capsule biopsies taken from stiff shoulder and elbow joints. These findings were consistent with the reports of Hildebrand *et al*. (10). Over time, the elevated cell number decreases, whereas the production of extracellular matrix increases. This process is potentially responsible for the development of joint contractures. Based on our established 3D collagen gel cell culture model, we simulated ECM-contraction *in vitro*. We demonstrate that MFs isolated from joint capsules not only exhibited α-SMA expression but also developed stress fibres, a hallmark of MFs. Compared to other mesenchymal cells (fibroblast cell line L929, osteoblasts), cultivated MFs demonstrated a relatively slow cell proliferation rate, which was verified by sporadic detection of Ki-67-positive cells (data not shown). Consequently, these cells are highly differentiated cells with a low proliferative potential. Our established 2D and 3D culture models enable the evaluation of the effects of various growth factors on the function of differentiated MFs. In serum-free medium, these growth factors diffuse into the collagen gel and directly influence the proliferation and differentiation of the cells. The use of serum-free medium is mandatory to eliminate the potential influence of growth factors in serum.

In our current study, we analyzed the influence of the growth factors TGF-β1 and PDGF on proliferation and ECM contraction. Both growth factors are released at high concentrations in the early stages of wound healing during the aggregation and activation of thrombocytes. TGF-β1 is the most potent and ubiquitous profibrogenic cytokine. Presumably, the growth factors induce proliferation and differentiation of local resting mesenchymal cells. We demonstrate that both growth factors induce proliferation and ECM contraction in fully differentiated MFs in a dose-dependent manner. In previous studies, the antifibrotic agent suramin competitively blocks the TGF-β and PDGF receptors [26, 27]. These results are consistent with our findings. Suramin significantly blocked TGF-β1- and PDGF-induced cell proliferation and collagen gel contraction, as well as TGF-β1- and PDGF-induced α-SMA gene expression and collagen type I gene expression. Furthermore, suramin blocked the down-regulatory effect of TGF-β1 on both TGF-β1 and TGF-β receptor 1 gene expression. Interestingly, the presence of suramin in our 3D MF culture without the addition of any growth factors resulted in a significant inhibition of ECM contraction, suggesting that MFs might have a basic level of TGF-β and PDGF production. In this context, we questioned whether MFs might have an alternative autoregulative potential.

During the initial inflammatory process after trauma, reactive oxygen species (ROS) are generated that can lead to tissue damage by affecting DNA, protein and lipid synthesis [33, 34]. Endogenous production of ROS can be induced by high concentrations of TGF-β1 [33]. The specific molecular targets of ROS are not yet identified, but their effects in cells have been noted in various physiological processes, including growth reduction, apoptosis, TGF-β1 autoinduction, activation of latent TGF-β1 molecules, induction of collagen synthesis and differentiation of MFs. We know from investigations of other organs (e.g., the lungs or the intestine) that ROS possess a profibrotic potential and induce collagen synthesis and fibroblast migration [28, 35]. ROS are regulated by the antioxidant system, which includes non-enzymatic catchers for free radicals as well as specific enzymes such as SOD and catalase.

To the best of our knowledge, there are no data available investigating the function of ROS on MFs in the development of posttraumatic joint contracture. In our current study, we detected a significant reduction of MF proliferation from the addition of the enzyme SOD itself, as well as a significant induction of ECM contraction without any significant impact on α-SMA or collagen type I gene expression in MFs. On the other hand, SOD blocked the contractile effect of TGF-β1. SOD did not reverse the down-regulation of TGF-β1 gene expression by TGF-β1. Furthermore, SOD had no significant impact on the gene expression of TGF-β receptors 1 and 2. These results indicate that TGF-β1 might regulate the function of MFs in joint capsules not only via a receptor signalling pathway but also by regulating the antioxidant system and ROS production. Furthermore, these observations support the theory that ROS are not only involved in the activation of MFs but also might influence their contractile function. The concentration of the TGF-β1 antibody used in our experiment was selected according to the study of Desmouliere *et al*. [25] and after several additional pre-tests. We investigated the effect of different concentrations of the TGF-β1 antibody on cell proliferation. The concentration of 5 μg/ml was an appropriate concentration to at least partially block the proliferative effect of TGF-β1 (data not shown). The intrinsic level of TGF-β1 expression is quite low in fully differentiated MFs. We did not expect any effect of the TGF-β1 antibody without additional extrinsic stimulation by TGF-β1. The purpose of the TGF-β1 antibody group was to be a specific internal control for the effect of TGF-β1. We did not intend to fully inhibit the TGF-β1 effect using the antibody.

In our study, we provide clear evidence that antifibrotic agents such as suramin and SOD are potent inhibitors of MF function in terms of both contractility and collagen I gene expression. Because most of the anti-oxidative agents are taken up by nutrition, it is of great interest to enhance our knowledge in this field. Many disorders are negatively influenced by food and nutrition. On the basis of an established 3D cell culture model, the cellular functions of MFs can be examined in depth to shed light on the mechanisms responsible for the development of post-traumatic joint capsule contractures. The model presented in our study may contribute to the development of new prophylactic and therapeutic strategies in joint capsule contracture as well as in other fibroconnective disorders. Further investigations on the potential use of antioxidant agents as therapeutic agents in post-traumatic contracture are warranted and could be tested in *in vivo* models in the future.
